# The Hippo pathway effector YAP inhibits HIF2 signaling and ccRCC tumor growth

**DOI:** 10.1038/s41421-022-00465-4

**Published:** 2022-10-06

**Authors:** Xu Li, Yong Suk Cho, Jian Zhu, Shu Zhuo, Jin Jiang

**Affiliations:** 1 Department of Molecular Biology, University of Texas Southwestern Medical Center, Dallas, TX USA; 2 Department of Pharmacology, University of Texas Southwestern Medical Center, Dallas, TX USA; 3 Present Address: Department of General Surgery, Central Hospital Affiliated to Shandong First Medical University, Shandong First Medical University & Shandong Academy of Medical Sciences, Jinan, Shandong China; 4 Present Address: Signet Therapeutics Inc, Research Institute of Tsinghua University in Shenzhen, Shenzhen, Guangdong China

**Keywords:** HIPPO signalling, Cancer therapy

Dear Editor,

Renal cell carcinoma (RCC) is among the top ten most diagnosed cancers around the globe. Clear cell renal cell carcinoma (ccRCC) makes up ~75% of renal malignancies and accounts for most of the renal cancer-associated death. ccRCC (> 90%) is mainly caused by loss-of-function mutations or deletion of the *Von Hippel-Lindau* (*VHL*) tumor suppressor gene, which results in stabilization and constitutive activation of Hypoxia-inducible factor 2α (HIF-2α) and ectopic expression of its target genes including those encoding glucose transporter type 1 (GLUT1) and vascular endothelial growth factor (VEGF) that regulate glycolysis and angiogenesis, respectively^1^.

The Hippo tumor suppressor pathway is an evolutionarily conserved signaling pathway that restricts tissue growth and regulates organ size by phosphorylating and inhibiting the activities of the pathway effectors YAP/TAZ^2,3^. In response to decreased Hippo signaling, unphosphorylated YAP/TAZ translocates into the nucleus and binds the TEAD-family of transcription factors to activate Hippo pathway target genes^4–6^. Aberrant activation of YAP promotes tumor progression in many types of cancers including liver, lung, breast, and gastrointestinal cancer^3^. Therefore, we were surprised to find that high YAP/TAZ expression levels correlate with good prognosis in ccRCC patients (Supplementary Fig. S1a, b). YAP expression levels were lower in ccRCC tumors than those in normal tissues and inversely correlated with ccRCC tumor grades (Supplementary Fig. S1c, d). Furthermore, YAP/TAZ expression levels were relatively low whereas YAP phosphorylation remained high in multiple *VHL* mutant ccRCC cell lines compared with *VHL* wild-type (WT) ccRCC or control cells (Supplementary Fig. S2). These observations raised an interesting possibility that high YAP/TAZ activity might be incompatible with ccRCC tumor growth. Indeed, treating ccRCC cells with XMU-MP-1, a small molecule inhibitor of Hippo/MST1/2 kinase that caused increased YAP nuclear localization (Supplementary Fig. S3)^7^, or expressing a constitutive active form of YAP (YAP-5SA) inhibited ccRCC cell growth in both 3D cultures and xenografts (Fig. 1a‒f; Supplementary Fig. S4). Consistent with a previous study^7^, XMU-MP-1 was well tolerated in mice: aside from reduced tumor weight and slightly increased spleen weight, both body weight and liver weight were normal (Supplementary Fig. S5). XMU-MP-1 inhibited HIF-2α target gene expression in ccRCC cells in a dose-dependent manner with little if any effect on HIF-2α protein level (Fig. 1g, h; Supplementary Fig S6), and this inhibitory effect was partially reversed by YAP/TAZ double knockdown (Fig. 1i, j). Of note, XMU-MP-1 did not inhibit the expression of HIF-1α target gene *PGK1* (Fig. 1h), consistent with the notion that HIF-2α but not HIF-1α is the oncogenic driver of *VHL*^*−/−*^ ccRCC^8^. RNA-seq experiments showed that HIF-2α target genes were enriched in genes downregulated by XMU-MP-1 in 786-O cells while YAP target genes were upregulated (Fig. 1k, l). In addition, YAP/TAZ double knockdown increased whereas overexpression of either WT YAP or YAP-5SA inhibited HIF-2α target gene expression (Fig. 1j, m). In contrast, TEAD-binding-deficient YAP variant (S94A) did not inhibit HIF-2α target gene expression and failed to suppress ccRCC tumor growth in xenografts (Fig. 1m; Supplementary Fig. S7). These results suggest that YAP inhibits HIF-2α transcriptional program and ccRCC cell growth through binding to TEAD.

**Fig. 1:**
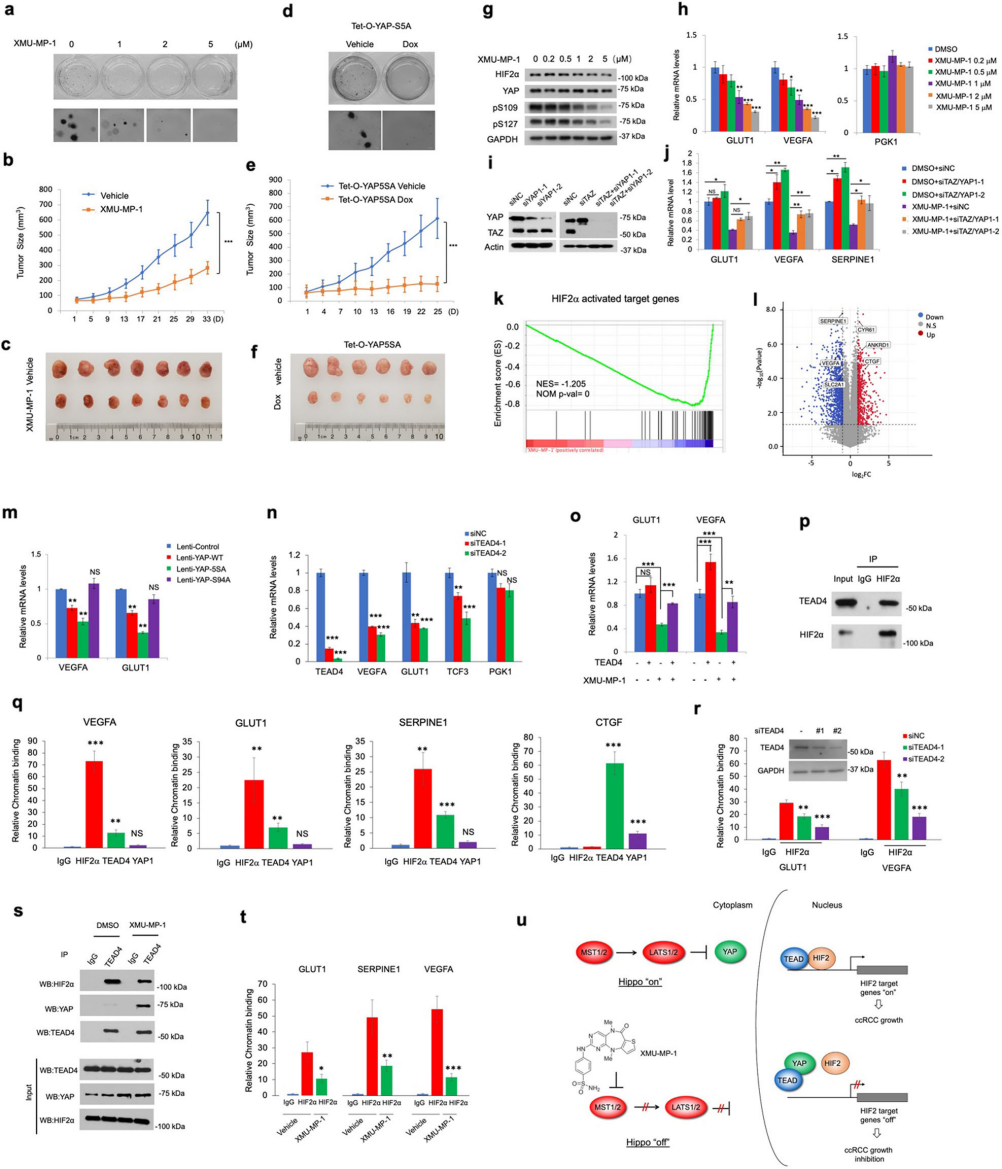
YAP inhibits HIF-2α and ccRCC tumor growth by disrupting the HIF-2α/TEAD signaling complex. **a** Anchorage-independent growth of 786-O cells treated with vehicle or XMU-MP-1 at the indicated concentrations. **b**, **c** Growth curves (**b**) and images (**c**) of 786-O xenograft tumors treated with vehicle or XMU-MP-1 for 33 days. *n* = 7 mice for each group. **d** Anchorage-independent growth of 786-O cells expressing Tet-O-YAP5SA and treated with vehicle or Doxycycline. **e**, **f** Growth curves (**e**) and images (**f**) of Tet-O-YAP5SA-expressing 786-O xenograft tumors treated with vehicle or Doxycycline. *n* = 6 mice for each group. **g**, **h** Protein levels of HIF-2α and YAP as well as levels of YAP phosphorylation on S109 and S127 were analyzed by the indicated antibodies (**g**) and relative mRNA levels of *GLUT1*, *VEGFA*, and *PGK1* were determined by RT-qPCR (**h**) in 786-O cells treated with XMU-MP-1 at the indicated concentrations. **i**, **j** Protein levels of YAP and TAZ (**i**) and relative mRNA levels of *GLUT1*, *VEGFA*, and *SERPINE1* (**j**) in 786-O cells treated with the indicated siRNAs in the absence or presence of 2 μM XMU-MP-1. Of note, the anti-YAP antibody recognized both YAP and TAZ on Western blot (WB). **k** Gene set enrichment analysis (GSEA) of RNA-seq data shows a depletion of HIF-2α target genes in 786-O cells treated with 2 μM XMU-MP-1. **l** Volcano plot shows the opposite effects of XMU-MP-1 treatment (2 μM) on HIF-2α target genes and the Hippo pathway signature genes in 786-O cells. **m** Relative mRNA levels of *VEGFA* and *GLUT1* in 786-O cells expressing the indicated YAP constructs. **n** Relative mRNA levels of the indicated genes in 786-O cells treated with the control (siNC) or two independent TEAD4 siRNAs (siTEAD4-1 and siTEAD4-2). **o** Relative mRNA levels of *VEGFA* and *GLUT1* in 786-O cells infected with or without TEAD4 lentivirus and treated with or without XMU-MP-1. **p** Co-IP experiment showing that HIF-2α forms a complex with TEAD4 in 786-O cells. **q** ChIP experiments showing that TEAD4 (but not YAP) and HIF-2α co-occupied on the promoter/enhancer regions of HIF-2α target genes while TEAD4 and YAP co-occupied on the promoter/enhancers region of a YAP target gene *CTGF*. **r** ChIP experiments showing that TEAD4 knockdown reduced HIF-2α binding to its target promoters/enhancers. **s** Co-IP experiment showing that treating 786-O cells with XMU-MP-1 reduced HIF-2α binding while increased YAP binding to TEAD4. Immunoprecipitates by IgG or anti-TEAD4 antibody (top) and cell extracts (bottom) were analyzed by WB with the indicated antibodies. **t** ChIP experiments showing that treating 786-O cells with XMU-MP-1 decreased HIF-2α binding to its target promoters/enhancers. **u** Model for how Hippo/MST1/2 inhibition or YAP activation inhibits HIF-2 transcriptional activity and ccRCC tumor cell growth (see text for details). Data in **h**, **j**, **m**, **n**, **o**, **q**, **r**, **t** are means ± SD. *P < 0.05, **P < 0.01, ***P < 0.001 (two-sided, unpaired *t*-test).

YAP forms a transcriptional complex with TEAD to regulate Hippo pathway target gene expression^4–6^; therefore, one would expect that YAP and TEAD should act in the same direction. However, high levels of TEAD4 correlated with poor prognosis in ccRCC patients (Supplementary Fig. S8a). In contrast to YAP, TEAD4 expression is higher in ccRCC tumors than that in normal tissue and positively correlates with ccRCC tumor grades (Supplementary Fig. S8b, c). Knockdown of either TEAD4 or TEAD1/3/4 inhibited HIF-2α target gene expression (Fig. 1n; Supplementary Fig. S9a, b) whereas overexpression of TEAD4 increased HIF-2α target gene expression and reversed the inhibitory effect of XMU-MP-1 (Fig. 1o). Furthermore, knockdown of TEAD1/3/4 inhibited ccRCC cell growth whereas overexpression of TEAD4 rescued ccRCC cell growth in the presence of XMU-MP-1 in 3D cultures (Supplementary Figs. S9c and S10), suggesting that TEAD4 is a positive regulator of HIF-2α target gene transcription as well as ccRCC cell growth. ChIP experiments showed that TEAD4 but not YAP co-binds with HIF-2α on the promoter/enhancer regions of multiple HIF-2α target genes (Fig. 1q). Co-IP experiments revealed that endogenous TEAD4 and HIF-2α formed a complex in ccRCC cells (Fig. 1p) and that exogenously expressed TEAD4 and HIF-2α formed a complex in HEK293T cells (Supplementary Fig. S11a). Furthermore, Knockdown of TEAD4 decreased the occupancy of HIF-2α on its target promoters/enhancers (Fig. 1r), suggesting that TEAD4 forms a complex with HIF-2α to promote HIF-2α binding to the promoter/enhancer regions of its target genes, thereby increasing the transcription of these genes.

Domain mapping revealed that TEAD4 interacted with HIF-2α through its C-terminal YAP-binding domain (Supplementary Fig. S11b, c), raising a possibility that YAP may compete with HIF-2α for binding to TEAD4. Indeed, Co-IP experiments showed that TEAD4‒HIF-2α interaction was inhibited by increasing amounts of YAP in HEK293T cells (Supplementary Fig. S11d). GST pull down assay using recombinant GST-TEAD4 fusion protein and Flag-tagged HIF-2α and YAP purified from HEK293A cells further demonstrated that YAP competed with HIF-2α for binding to TEAD4 (Supplementary Fig. S11e). TEAD4‒HIF-2α interaction was also inhibited by the blockage of Hippo signaling with XMU-MP-1 in both HEK293T and 786-O cells (Fig. 1s; Supplementary Fig. S11f). In addition, XMU-MP-1 inhibited the binding of HIF-2α to its target promoters/enhancers (Fig. 1t). Finally, YAP5SA but not YAP5SAS94A inhibited TEAD4‒HIF-2α interaction, their binding to HIF-2α target promoters/enhancers, and the expression of HIF-2α target genes (Supplementary Fig. S12), suggesting that YAP‒TEAD4 interaction blocks TEAD4‒HIF-2α complex formation and their cooperative binding to HIF-2α target promoters/enhancers.

Taken together, we propose the following working model (Fig. 1u). TEAD physically interacts with HIF-2α to enhance its promoter/enhancer occupancy through cooperative binding, leading to enhanced HIF-2α target gene expression. Hippo pathway inhibition increases nuclear YAP, which inhibits HIF-2α promoter/enhancer occupancy by competing with HIF-2α for the same pool of TEAD, leading to decreased HIF-2α target gene expression and impediment of ccRCC tumor growth. Indeed, TEAD levels were relatively low in ccRCC cell lines so that it might be possible that TEAD becomes less accessible to HIF-2α when nuclear YAP increased (Supplementary Fig. S13). Finally, we found that Hippo pathway inhibition by XMU-MP-1 did not significantly upregulate most of the YAP target genes involved in cell proliferation in 786-O cells (Supplementary Fig. S14), which could explain why XMU-MP-1 did not stimulate ccRCC tumor growth. The failure of activating YAP oncogenic program by XMU-MP-1 in ccRCC could be due to the relatively low expression of YAP/TAZ/TEAD and possibly, other coregulators in these cells. In addition, high levels of HIF-2α in ccRCC may further limit the accessibility of TEAD to YAP.

The prevalent view in the Hippo field is that Hippo signaling inhibits tumor growth by blocking the oncogenic potential of YAP. Our study has uncovered a noncanonical mechanism by which YAP acts through TEAD to regulate cancer cell growth. In the conventional model, YAP forms a transcriptional complex with TEAD to regulate the expression of cancer related genes^3^. Here, we revealed an antagonistic relationship between YAP and TEAD in the regulation of ccRCC progression. We found that TEAD acts as a critical cofactor for the oncogenic driver HIF-2 to activate GLUT1 and VEGF, and this function of TEAD is antagonized by nuclear YAP (Fig. 1u). The antagonistic relationship between YAP and TEAD has also been implicated in the regulation of estrogen receptor (ER)-positive breast cancer and androgen receptor (AR)-positive prostate cancer^9,10^, suggesting that the mechanism we uncovered here could be extended to other tumors. Interestingly, a recent study showed that elevated YAP/TAZ in LATS1/2 KO mice interfered with HIF-1α function during hypoxia-induced bone angiogenesis^11^, suggesting that YAP could also inhibit HIF-1α although the underlying mechanism remains undetermined. Of note, because HIF-2 is not the only factor that contributes to *VHL*^*−/−*^ ccRCC tumor growth, it remains possible that YAP could inhibit ccRCC progression through additional mechanism(s).

ccRCC is a deadly disease. Therapies targeting HIF-2α and its downstream effectors such as VEGF are the standard of care or in clinic trials; however, drug resistance occurs in most patients, making it necessary and urgent to develop new therapeutics^12^. Our findings that Hippo pathway inhibition or YAP activation can inhibit HIF2α signaling and ccRCC tumor growth open an exciting possibility for developing novel therapeutics to treat ccRCC by targeting the Hippo-YAP-TEAD-HIF-2α signaling axis.

## Supplementary information

Supplementary information
