# Supplementary material for: The Hippo pathway effector YAP inhibits HIF2 signaling and ccRCC tumor growth
Source: Cell Discov. 2022 Oct 6;8:103. doi: 10.1038/s41421-022-00465-4 (PMC9537283; doi:10.1038/s41421-022-00465-4)
Supplement: Supplementary file 1 — Supplementary information [file 41421_2022_465_MOESM1_ESM.pdf]

Supplementary information

Materials and Methods

DNA constructs

Full length HIF-2α construct was amplified by PCR and the PCR products were sub-cloned into a pCMV-6xMyc vector. The YAP-WT and YAP-5SA constructs were described previously[13]. YAP-S94A and YAP-5SAS94A were generated from YAP-WT and YAP-5SA by PCR-based mutagenesis. Lenti-TEAD4-C-MYC-DKK construct was purchased from Origene (Cat. No. RC219686L1). TEAD4 was cloned into FUGW-UBC-GFP vector to generate FUGW-UBC-Flag-TEAD4 construct. YAP-WT or YAP-5SA was cloned into the pLVX-IRES-ZsGreen vector. Coding sequences for GFP, YAP5SA and YAP5SAS94A were subcloned into pTet-O-Ngn2-puro (Addgene plasmid, Cat. No 52047) to generate Tet-O-GFP, Tet-O-YAP5SA and Tet-O-YAP5SAS94A. pLenti-CMV-rtTA3 Hygro (Addgene plasmid, Cat. No. 26730) was used to generate Dox-inducible cell lines.

Cell cultures

786-O, 769-P, A498, RCC4, UMRC2, UMRC6, Caki-1, and HKC are maintained with RPMI-1640 (Gibco, Cat. No. 42401018) supplemented with 2 mM L-glutamine (Gibco, Cat. No. 25030081) and 10% FBS. HEK293T and HEK293A cells are cultured with high glucose Dulbecco's Modified Eagle's Medium that contains 4.5 g/L glucose and 4 mM L-glutamine (DMEM, Gibco, Cat. No. 11965092) supplemented with 10% Fetal Bovine Serum (FBS, Gibco, Cat. No. 16000044).

Immunoblot analysis

Cells were harvested and lysed with lysis buffer containing 1M Tris pH8.0, 5M NaCl, 1M NaF, 0.1M Na_3VO_4, 1% NP-40, 10% glycerol, and 0.5M EDTA (pH 8.0). Proteins were separated by electrophoresis on SDS-polyacrylamide gel electrophoresis (PAGE) and electro-transferred to PVDF membrane. Membranes were washed with TBST and incubated with primary antibodies for 2 hours. And then the membranes were washed for three times with TBST and incubated with second antibodies for 2 hours, after washed for three times with TBST, the membranes were probed with ECL system (Cytiva, Cat. No. RPN2105). The antibodies used in this study were listed here: mouse anti-YAP (Santa Cruz, Cat. No. sc-101199); mouse anti-TAZ (Santa Cruz Biotechnology, catalog # sc-293183); rabbit anti-P-YAP(S127) (Cell Signaling, catalog # 13008); rabbit anti-P-YAP(S109)(Cell Signaling, catalog #53749); rabbit anti-HIF-2α (BETHYL, Cat. No. A700-003); mouse anti-TEAD4 (Santa Cruz, Cat. No. sc-101184); rabbit anti-Pan-TEAD(Cell Signaling, catalog #13295); rabbit anti-HA (COVANCE, Cat. No. MMS-101R); mouse anti-Myc (Santa Cruz, Cat.No.SC-40); anti-Myc (Abcam, Cat. No. ab32); anti-Myc (Abcam, Cat. No. Ab9106); anti-Flag (Sigma, Cat. No. F3165); anti-GFP (Abcam, Cat. No. ab290). Peroxidase-Conjugated AffiniPure Goat Anti-Mouse IgG (Jackson ImmunoResearch Code,115-035-003) or Goat Anti-Rabbit IgG (Jackson ImmunoResearch, Code,111-035-144). Chemiluminescent signals were visualized with ECL system (Cytiva, Cat. No. RPN2105).

Immunofluorescence assay

786-O cells were fixed with 4% paraformaldehyde in PBS for 10 min, permeabilized with 0.2% Triton X-100 (Sigma, Cat. No. T8787) for 5 min and blocked by 5% BSA in PBS for 1 h. A rabbit anti-HIF-2α antibody (BETHYL, Cat. No. A700-003) and mouse anti-YAP monoclonal antibody (Santa Cruz, Cat. No. SC-101199) were used, followed by Cy2- and Cy3-conjugated secondary antibodies (Jackson ImmunoResearch). As negative controls, the samples were incubated with the secondary antibodies without primary antibodies. Images were captured by Zeiss LSM510

confocal microscopy. The acquired pictures were further processed and assembled using ImageJ.

Co-immunoprecipitation assay

Immunoprecipitation was performed according to standard protocol. 786-O cell lysates were incubated with antibodies or mouse IgG for overnight at 4℃, followed by immobilization and precipitation with Protein A resin. The bound proteins were analyzed by western blot. For overexpression experiments, HEK293T cells were transfected with 5μg myc-HIF-2α and GFP-TEAD4 plasmids in the absence or presence of the Flag-YAP construct in 10 cm dishes. Cell lysates were incubated with antibodies against epitope tags for overnight, followed by immobilization and precipitation with Protein A resin. The bound proteins were analyzed by immunoblot assay. Antibodies used for IP experiments: anti-HIF-2α (BETHYL, Cat. No. A700-003), anti-TEAD4 (Santa Cruz, Cat. No. sc-101184), anti-Flag (Sigma, Cat.No. F3165), anti-Myc(Santa Cruz, Cat.No.SC-40), anti-GFP (Abcam, Cat. No. ab290), and mouse IgG (Santa Cruz, Cat.No. SC-3881).

GST pull down assay

GST-TEAD4 construct was generated by subcloning the full-length human TEAD4 into the pGEX-4T1 vector (Sigma-Aldrich). GST-TEAD4 fusion protein was expressed in *E. coli* BL21 (DE3) and purified using glutathione-4B Sepharose (GE Healthcare) at 4°C. Bacteria suspension in TBS (50 mM Tris-Cl, pH 7.4, 150 mM NaCl) was extracted for 5 min by sonication. After centrifuging at 12,000xg for 1hr, the supernatant was incubated with glutathione-4B Sepharose for 1 hour. with rotation. After washing with TBS twice, GST-TEAD4 was eluted with 50 mM GSH. For purification of Flag-YAP and Flag-HIF2α, HEK-293A cells were transfected with the Flag-YAP and Flag-HIF2α constructs, respectively, grown for 48 hours, and harvested in lysis buffer (1M Tris pH8.0, 5M NaCl, 1M NaF, 0.1M Na_3VO_4, 1%

NP-40, 10% glycerol). Cell Extracts were incubated with M2 (anti-Flag) antibody (Sigma-Aldrich) overnight at 4°C, followed by immobilization and precipitation with Protein A resin (Pierson) for 2 hours at 4°C. The Immunoprecipitants were then washed and bound proteins were eluted with Flag peptide (Sigma-Aldrich) according to the manufacturer's instructions. Flag-YAP and Flag-HIF2α were incubated with purified GST-TEAD4 for overnight at 4°C, followed by immobilization and precipitation with glutathione-4B Sepharose for 2 hours at 4°C. The bound proteins were analyzed by immunoblot assay with anti-Flag (Sigma, Cat.No. F3165) and anti-GST (Santa Cruz, Cat. No. sc-138) antibodies.

RNA interference

For RNAi experiments in renal cancer cells, the siRNAs were acquired from the Sigma-Aldrich. The RNAi MAX reagent (Invitrogen Cat. No. 13778150) was used for the transfection of siRNA according the manuscription. Knockdown efficiency was validated by RT-PCR and/or immuno-blotting. The sequences for YAP silencing were: 5'- CAC CUA UCA CUC UCG AGA U-3' and 5'-GCU CAU UCC UCU CCA GCU U-3'. The sequences for TAZ silencing were: 5'-CCC AGA CAU GAG AUC CAU CAC UAA U -3'. The sequences for TEAD4 silencing were: 5'- CAG AGU AUG CUC GCU AUG A -3' and 5'- CUC GCU AUG AGA AUG GAC A -3'. The sequences for TEAD1/3/4 silencing were: 5'- AUG AUC AAC UUCA UCC ACA AG -3' and 5' GAU CAA CUU CAU CCA CAA GCU -3'. The sequences for negative control were: 5'-UUC UCC GAA CGU GUC ACG U-3'.

Virus infection and transient transfection

For packaging lentivirus, HEK293T cells were transfected with the expression vectors and package vectors (psPAX2 and pMD2.G) by PolyJet (SignaGen laboratories, Cat. No. SL100688). After 48 hours, the supernatants of the medium were collected and filtered with 0.45 μm filter. The supernatant containing virus was stored in 4°C for cell infection. The renal cancer

cells were cultured in fresh media and subsequently infected with lentivirus overnight together with Polybrene (Sigma, Cat. No. H9268). Hygromycin B (Sigma, Cat. No. 10843555001) and Puromycin (Sigma, Cat. No. P9620) were used for infected cells selection according to the resistance of the vectors.

RNA extraction and RT-qPCR analysis

We extracted the total RNA by RNeasy plus mini kits according to the protocol (Qiagen, Cat. No. 74106). After RNA extraction, the RNA was subjected to reverse transcription PCR for cDNA synthesis according to the RT-PCR kit (Applied Biosystems, Cat. No. 4368814). The relative gene expression was measured according to $2^{-\Delta\Delta CT}$ methods. The house keeping gene 36B4 was used for internal control. The Primer sequences were: 36B4, F: GGC GAC CTG GAA GTC CAA CT; R: CCA TCA GCA CCA CAG CCT TC; GLUT1, F:TGG CAT CAA CGC TGT CTT CT, R:CTA GCG CGA TGG TCA TGA GT; VEGFA, F:CTT GCA GAT GTG ACA AGC CG, R:GTC GAT GGT GAT GGT GTG GT; SERPINE1, F: ACA ACC CCA CAG GAA CAG TC; R: GAT GAA GGC GTC TTT CCC CA; PGK1, F: ATG CTT TTG GCA CTG CTC AC, R: ACT TTA GCT CCG CCC AGG AT; TCF3, F: CAC GGC CTG CAG AGT AAG AT, R: CCC TAG CCC ACT GTA GGA GT; TEAD4, F: GAA CGG GGA CCC TCC AAT G, R: GCG AGC ATA CTC TGT CTC AAC

ChIP qPCR

ChIP (Chromatin Immuno-precipitation) assay was performed as previously study description[14], In brief, cells were cross-linked with 1% formaldehyde for 10min and quenched by adding glycine. For TEAD and YAP ChIP assay, cells were cross-linked using 2 mM DSG crosslinker (CovaChem, Cat. No.13301) at room temperature for 1 h, followed by secondary fixation with 1% formaldehyde (Pierce, Cat. No. 28908) for 10 min and quenched by glycine. Subsequently, cells were washed with cold PBS and subject to cell lysis. The cell extracts were sonicated by

Bioruptor. After centrifuge, the supernatants were incubated with prepared HIF-2α/TEAD antibody-Dynabeads for 1 hour at room temperature and another 1 hour at 4℃. The slurries were washed in wash buffer for 5 times and de-cross-linked ChIP in elution buffer at 65℃ overnight. The enriched DNA was extracted via DNA extraction kits (Qiagen, Cat. No. 28106) and subject to quantitative PCR analysis. The Primer sequences for ChIP-qPCR were: GLUT1,F: GGA TGC TGG AGC CTA GTG TG, R: CCA TCA CGG TCC TTC TTC ATG; VEGFA, F: GCC AGA CTC CAC AGT GCA TA, R: TCT GGA GCT GCT AGG ACC C; SERPINE1, F: ACA ACC CCA CAG GAA CAG TC, R: GAT GAA GGC GTC TTT CCC CA; CTGF, F: TGT GCC AGC TTT TTC AGA CG, R: TGA GCT GAA TGG AGT CCT ACA CA. The antibodies used in ChIP-qPCR were anti-HIF-2α (NOVUS, Cat. No. NB100-122), anti-TEAD4 (Santa Cruz, Cat. No. SC-101184) and anti-YAP (Santa Cruz, Cat. No. SC-271134). Anti-rabbit IgG dynabeads (Invitrogen, Cat: 11204D) and anti-mouse IgG dynabeads (Invitrogen, Cat. No. 11031) were used to bind antibodies.

RNA sequencing and data analysis

The global gene expression analysis (Vehicle vs XMU-MP-1 treated groups) was based on RNA sequencing platform from BGI (Beijing Genomic Institute).Cellular RNA was extracted using Qiagen RNA extraction kit (Qiagen; Cat: 74104) according to the manufacturer's instructions. The cellular RNA was sent to BGI Genomics (https://www.bgi.com) for RNA sequencing. RNA was quality-accessed with an Agilent 2100 Bioanalyzer (Agilent RNA 6000 Nano Kit) with RNA integrity number above 9 for library construction. The total RNA was used for library construction according to the protocol of BGISEQ-500 platform. The libraries were sequenced using BGISEQ-500 platform. Then the FASTQ sequencing files were aligned to the hg19 human genome using STAR aligner with uniquely mapped reads kept for further analysis. Differential expression was analyzed using DESeq2 with default parameters. The RNA sequence data are

deposited in the Gene Expression Omnibus (GEO) database (Assessing number: GSE197468). For gene set enrichment analysis of RNA-seq data, gene sets of HIF2A activated target genes was used and downloaded from Molecular Signatures Database v7.4, GSEA was implemented using the GSEA 4.1.0 software, with default parameters. Volcano plot of DEGs (Threshold $P<0.01$ and fold change>2) was generated using the OmicStudio tools (https://www.omicstudio.cn/tool).

Analysis of TCGA kidney renal clear cell carcinoma data sets

Analysis of YAP, TAZ and TEAD4 expression and correlation with patient survival time in kidney renal clear cell carcinoma TCGA data sets were performed with UALCAN online analysis platform (http://ualcan.path.uab.edu/analysis.html).

Anchorage-Independent Growth Assays

Cells were plated at a density of 5000 cells/mL for 786-O, UMRC6, A498 and 769-P cells in complete medium with 0.4% agarose onto bottom layers composed of medium with 1% agarose. Cells were incubated at 4ºC for 10 min and then placed in a 37ºC incubator. Every 4 days, three drops of complete media were added onto the plate. After incubating cells for 2 to 4 weeks, extra liquid on the plate was aspirated and colonies were stained with 100 µg/mL iodonitrotetrazolium chloride (Sigma, Cat.No.I8377) in growth medium. Cell culture plates were incubated overnight prior to analysis.

Xenograft tumor models

The procedures for all animal experiments were reviewed and approved by the IACUC of UT Southwestern Medical School. Six-week-old male NOD scid gamma (NSG) mice were used and tumor cells were implanted subcutaneously into the dorsal flank of the mice (1×10^6 cells suspended in 100ul PBS with 50% Matrigel). When tumor xenografts reached a mean volume of

~100 mm^3 (length × $width^2$ /2), mice were randomly assigned to experimental treatment groups (6-8 mice/ group). XMU-MP-1 (dissolved in 5% dextrose in water) was administered intraperitoneally twice daily with 10mg/kg body weight for the indicated time periods, and the control group were injected with the solvent. Dox in PBS was injected intraperitoneally with 20mg/kg body weight every day. Tumor size was measured using digital calipers. At the end of the studies, mice were sacrificed, and liver, spleen and tumors were harvested and weighed.

Statistics and reproducibility

All experiments were performed at least three independent times unless noted. Two-sided, unpaired *t*-test was used for comparisons. A P-value of < 0.05 was considered to be significant. Error bars on the graphs were presented as the s.d.

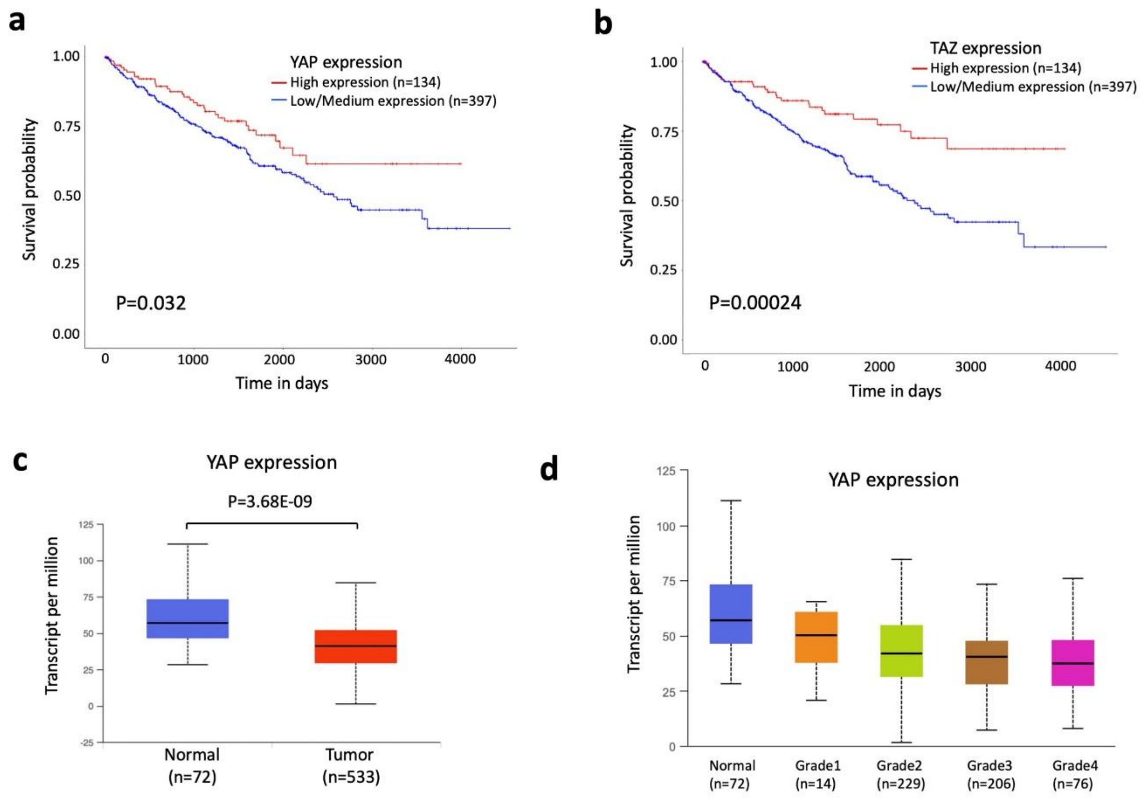

Fig. S1. High YAP/TAZ expression correlates with good prognosis in ccRCC patients

a, b Kaplan-Meier graph of overall survival shows that high YAP (**a**) or TAZ (**b**) favors the survival in ccRCC patients. **c** Analysis of TCGA database shows that mRNA levels for YAP are lower in ccRCC cancer samples (n=533) compared with normal tissues (n=72). **d** Analysis of TCGA database shows that mRNA levels for YAP are progressively lower in higher grades of ccRCC.

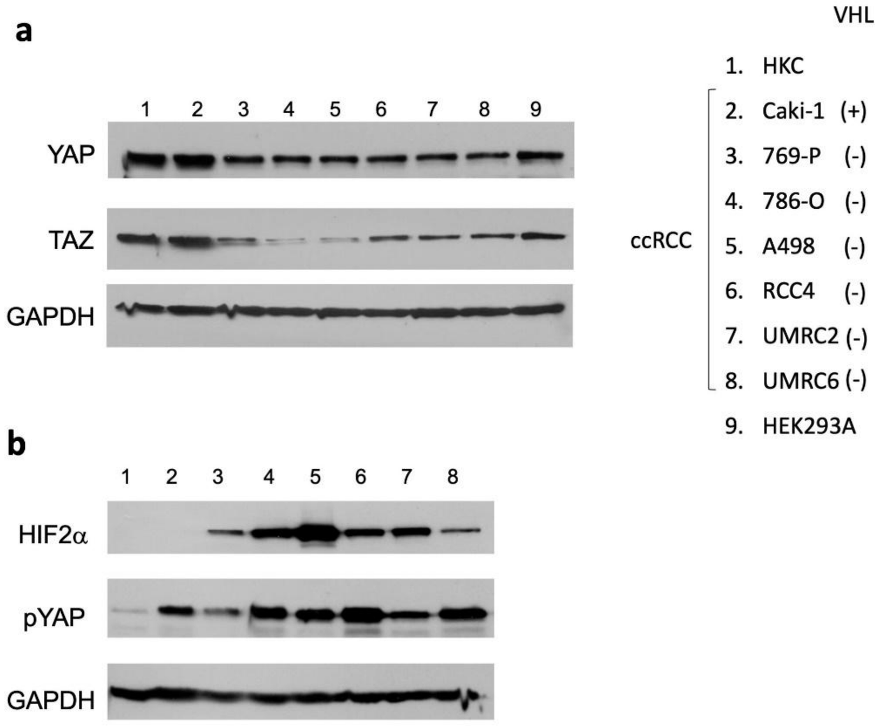

Fig. S2. YAP/TAZ expression is relatively low in *VHL* mutant ccRCC cell lines

a, b Western blot analysis of YAP/TAZ protein expression (**a**) and YAP phosphorylation (pS127) and HIF-2α expression (**b**) in the indicated cell lines. YAP/TAZ protein levels are lower in *VHL* mutant (-) ccRCC (lane 3-8 in **a**) compared with VHL wild type (+) ccRCC (Caki-1 or control cell lines HKC and HEK293A.

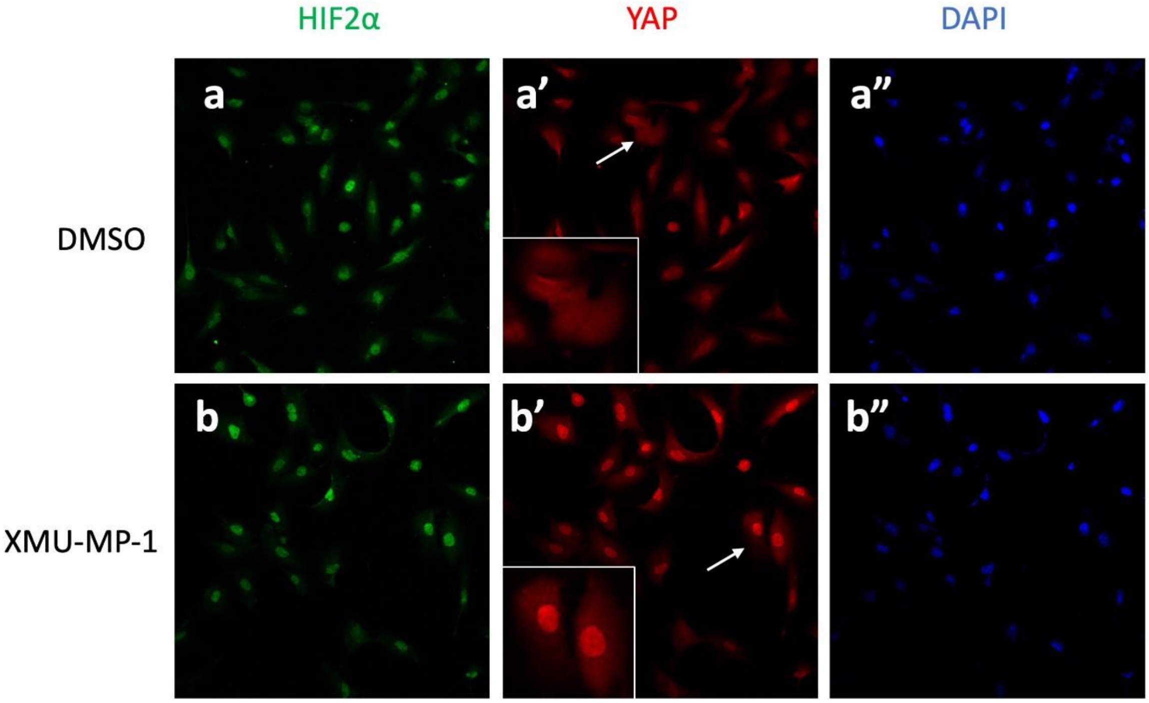

Fig. S3. XMU-MP-1 induced YAP nuclear accumulation in 786-O cells

a, b 786-O cells were treated with vehicle (a-a”) or XMU-MP-1 (b-b”) and immunostained for HIF-2α (green), YAP (red) and the nuclear marker DAPI (blue). Insets are large magnification of cells indicated by the arrows in **a’** and **b’**.

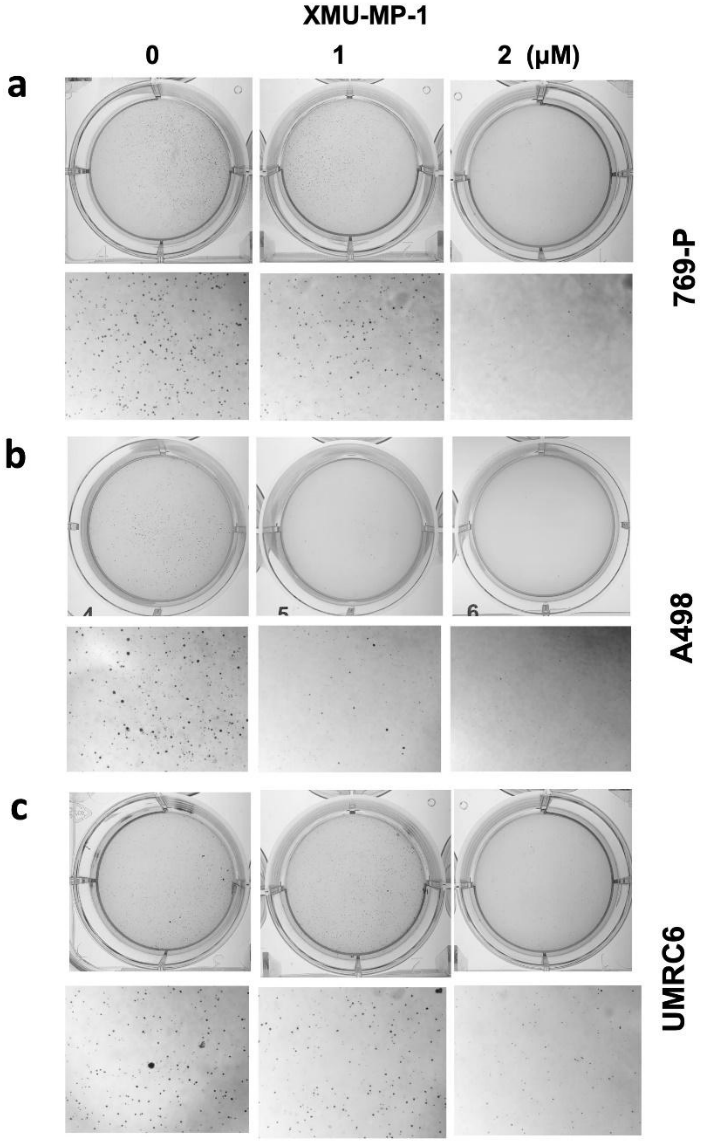

Fig. S4. XMU-MP-1 inhibited anchorage-independent growth of multiple ccRCC cell lines

a-c Anchorage-independent growth of ccRCC cells 769-P (**a**), A498 (**b**), and UMRC6 (**c**) in the absence or presence of XMU-MP-1 at the indicated concentrations. XMU-MP-1 inhibited ccRCC cell growth in a dose dependent manner.

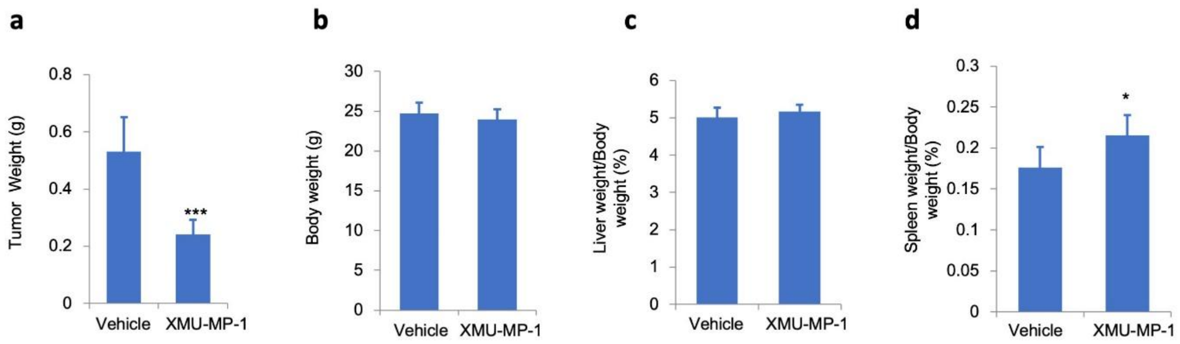

Fig. S5. XMU-MP-1 is well-tolerated in mice

a-d Tumor weight (**a**), body weight (**b**), liver weight/body weight (**c**) and spleen weight /body weight (**d**) of mice bearing 786-O tumors and treated twice daily with vehicle or XMU-MP-1 for 33 days. Data are means ± SD. $^{*}P<0.05$, $^{***}P<0.001$ (two-sided, unpaired *t*-test). n=7 mice for each group.

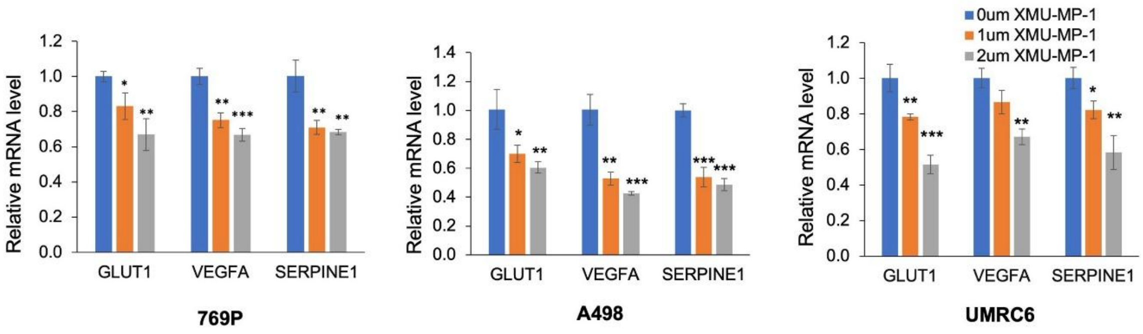

Fig. S6. XMU-MP-1 inhibited target gene expression in multiple ccRCC cell lines

Relative mRNA expression of HIF-2α target genes in the indicated ccRCC cells treated with vehicle or XMU-MP-1 at the indicated concentrations. XMU-MP-1 inhibited the expression of HIF-2α target genes in a dose dependent manner. Data are means ± SD. $^{*}P<0.05$, $^{**}P<0.01$, $^{***}P<0.001$ (two-sided, unpaired *t*-test).

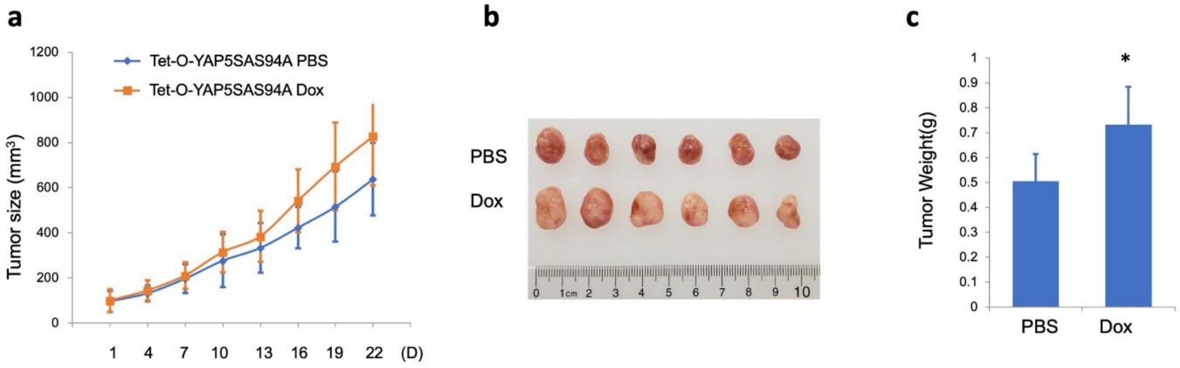

Fig. S7. YAP-5SAS94A did not inhibit 786-O tumor growth *in vivo*

a-c Female NOD scid gamma (NSG) mice bearing 786-O tumors that express Tet-O-YAP-5SAS94A were injected i.p. with PBS containing Dox (20mg/kg) or PBS daily for the indicated period. Tumor growth curve (**a**), photograph of tumor samples (**b**), and quantification of tumor weight (**c**) at the end of treatment were shown. Data are means ± SD. *P<0.01 (two-sided, unpaired *t*-test).

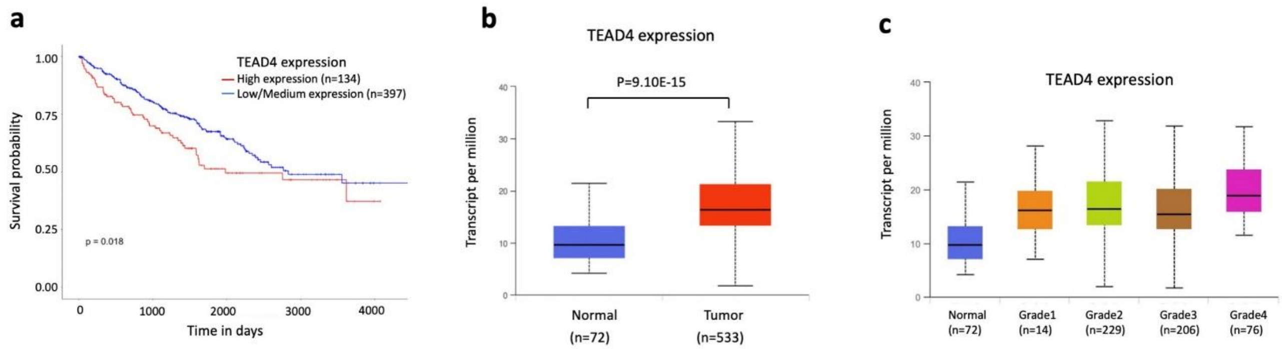

Fig. S8. High TEAD4 correlates with poor prognosis in ccRCC patients

a, Kaplan-Meier graph of overall survival shows that high TEAD4 correlates with poor prognosis in ccRCC patients. **b** Analysis of TCGA database shows that mRNA levels for TEAD4 are lower in ccRCC cancer samples (n=533) compared with those in normal tissues (n=72). **c** Analysis of TCGA database for TEAD4 mRNA levels in ccRCC of the indicated tumor grades.

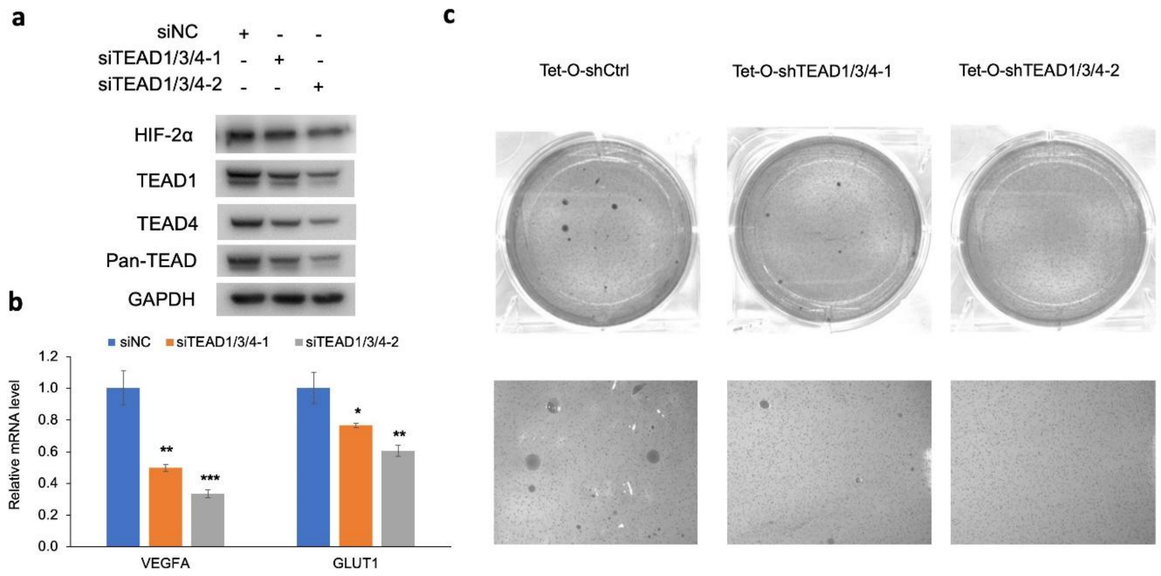

Fig. S9. TEAD knockdown affected HIF-2α target gene expression and ccRCC growth

a Western blot analysis showing the protein expression of HIF-2α, TEAD1, TEAD4 and Pan-TEAD in 786-O cells treated with control siRNA or two independent siRNAs targeting TEAD1/3/4. **b** Relative mRNA levels of VEGFA and GLUT1 in 786-O cells treated control shRNA or two independent shRNAs targeting TEAD1/3/4. Data are means ± SD. *P<0.05, **P<0.01, ***P<0.001 (two-sided, unpaired *t*-test). **c** Anchorage-independent growth of 786-O cells treated control siRNA or two independent siRNAs targeting TEAD1/3/4.

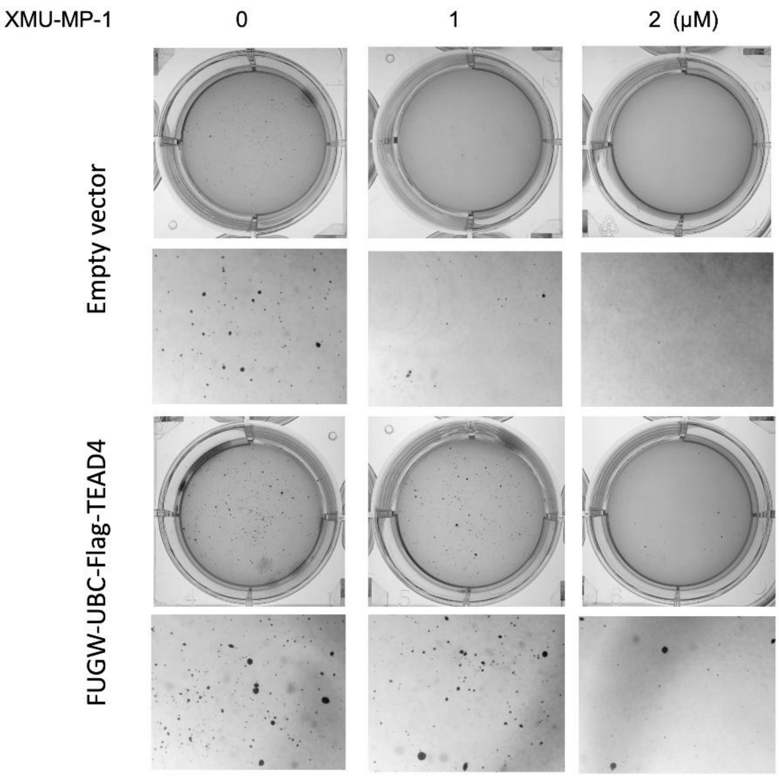

Fig. S10. TEAD4 overexpression rescued ccRCC growth in the presence of MST1/2 inhibitor

Anchorage-independent growth of control 786-O cells expressing empty vector (top) or 786-O cells expressing FUGW-UBC-Flag-TEAD4 (bottom) treated with XMU-MP-1 at the indicated concentrations. Overexpression of TEAD4 allowed 786-O cells to grow when MST1/2 was inhibited by XMU-MP-1.

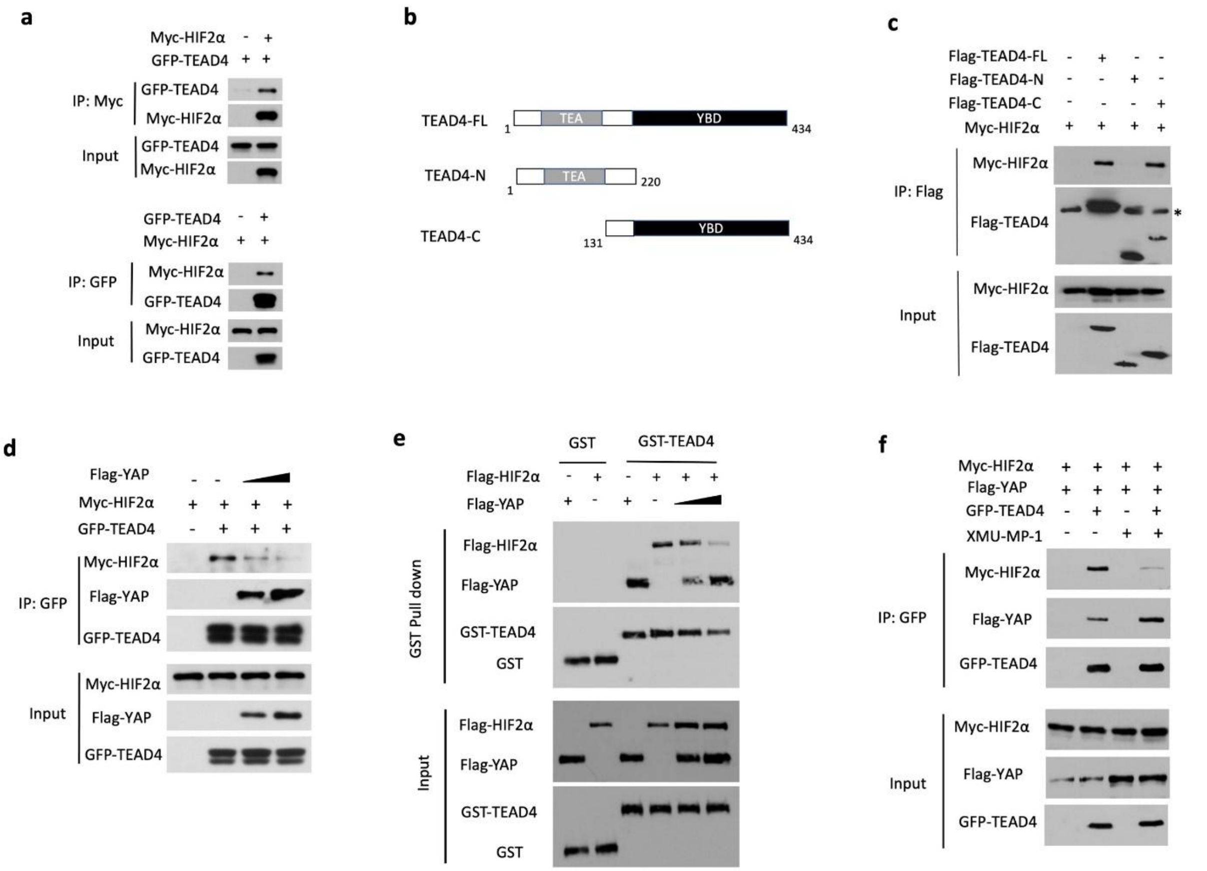

Fig. S11. YAP overexpression or MST1/2 inhibition disrupted HIF-2α/TEAD4 interaction

a Exogenously expressed HIF-2α and TEAD4 formed a complex in HEK293A cells. HEK293A cells were transfected with Myc-HIF-2α and GFP-TEAD4-expressing constructs. Cell extracts were immunoprecipitated with either anti-Myc (top) or anti-GFP (bottom) antibody, followed by western blot analysis with the indicated tag antibodies. **b** Diagrams of the indicated TEAD4 constructs with DNA binding domain (TEA) and YAP binding domain (YBD) indicated by gray and black boxes, respectively. **c** HIF-2α binds C-terminal region of TEAD4. HEK293A cells were transfected with Myc-HIF-2α and indicated Flag-TEAD4- expressing constructs. Cell extracts were immunoprecipitated with anti-Flag antibody, followed by western blot analysis with the indicated tag antibodies. **d** YAP disrupted HIF-2α/TEAD4 interaction. HEK293A cells were transfected with Myc-HIF-2α and GFP-TEAD4-expressing constructs without or with increasing

amount of Flag-YAP construct. Cell extracts were immunoprecipitated with anti-GFP antibody, followed by western blot analysis with the indicated tag antibodies. **e** YAP inhibited HIF-2α/TEAD4 interaction *in vitro*. GST control and GST-TEAD4 fusion proteins were incubated with immunopurified Flag-HIF-2α, Flag-YAP, or both, followed by western blot analysis of Flag-HIF-2α and Flag-YAP pulled down by GST-TEAD4. **f** MST1/2 inhibition disrupted HIF-2α/TEAD4 interaction while increasing YAP/TEAD4 interaction. HEK293A cells were transfected with the indicated constructs and treated with vehicle or XMU-MP-1. Cell extracts were immunoprecipitated with anti-GFP antibody, followed by western blot analysis with the indicated tag antibodies.

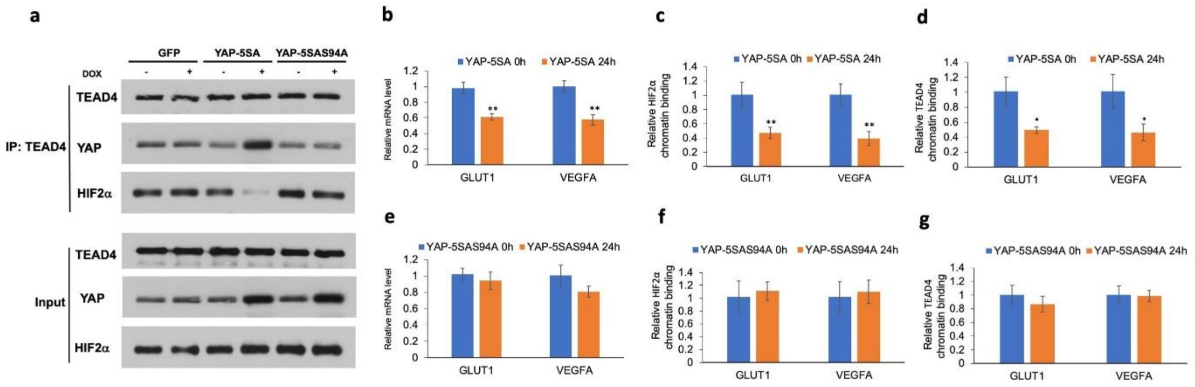

Fig. S12. YAP-5SA blocked HIF-2α/TEAD interaction and co-binding on HIF-2α target promoters/enhancers

(**a**) YAP-5SA but not YAP-5SAS94A inhibited HIF-2α/TEAD4 interaction. 786-O cells stably expressing Tet-O-GFP, Tet-O-YAP-5SA or Tet-O-YAP-5SAS94A were treated with 0.2 μg/ml doxycycline (Dox) for 24 hours, followed by IP and western blot analyses with the indicated antibodies. (**b-g**) YAP-5SA but not YAP-5SAS94A inhibits HIF-2α target gene expression and the binding of HIF-2α/TEAD4 to HIF-2α target promoters/enhancers. 786-O cells stably expressing Tet-O-YAP-5SA (**b-d**) or Tet-O-YAP-5SAS94A (**e-g**) were treated with 0.2 μg/ml doxycycline (Dox) for 0 or 24 hours, followed by RT-qPCR (**b, e**) and ChIP-qPCR analyses (**c, d, f, g**). Data are means ± SD. *P<0.05, **P<0.01 (two-sided, unpaired *t*-test).

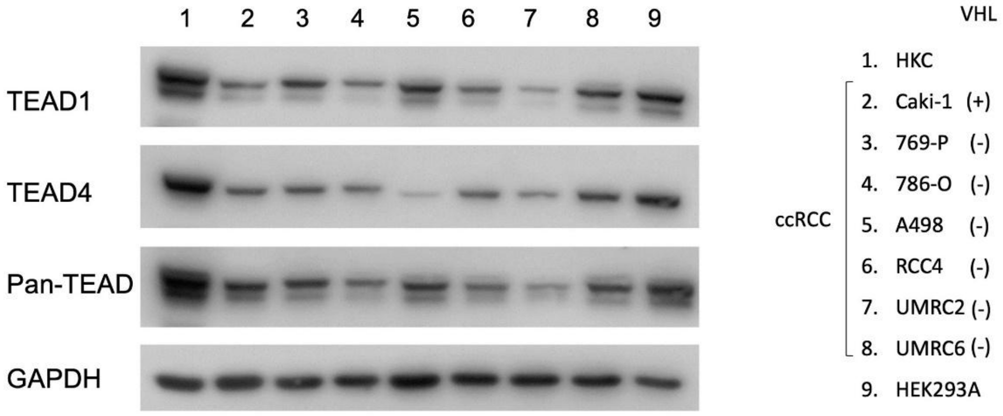

Fig. S13. TEAD expression levels are relatively low in ccRCC cell lines

Western blot analysis of TEAD protein expression using anti-TEAD1, anti-TEAD4, and anti-pan-TEAD antibodies in the indicated cell lines. TEAD protein levels were lower in ccRCC cells (lane 2-8) compared with control cell lines HKC (lane 1) and HEK293 (lane 9).

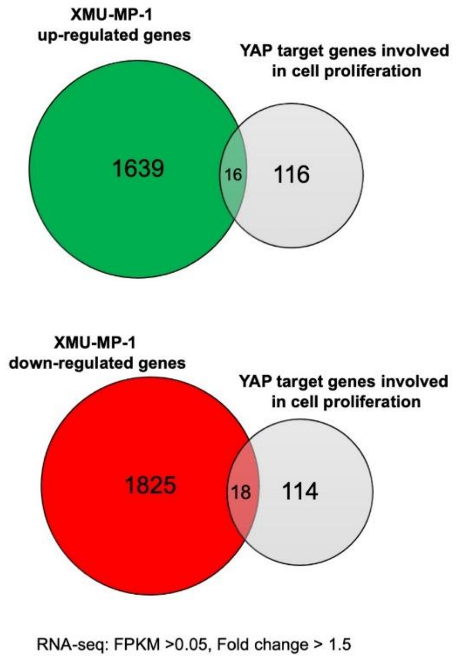

YAP target genes involved in cell proliferation

CTGF	MRE11	THOC1	CDC25A	AJUBA	GADD45GIP1	PPIF
CYR61	SERTAD2	ZWILCH	RND3	LSG1	MAP3K1	UTP20
HBEGF	ERCC6L	TIMM8A	RCC1	GTPBP4	PAWR	DKC1
RRM2	ODC1	SUV39H2	RBL1	TDP1	HSPA9	FGF5
THOC6	KIF20B	SMC3	KIF14	ADRB2	BUB1B	ETS1
EID2	NCBP2	RABGEF1	CENPF	NUP93	TRDMT1	RRS1
CDC6	TUBB6	CDH4	XPO6	TUBB	BOD1	SERPINE1
GADD45B	KNTC1	POLA2	CCNA2	PSRC1	GNL3	BCAT1
RAB11FIP1	NUP188	E2F3	RAD18	RUVBL2	ZPR1	FOSL1
NASP	TNFRSF12A	EIF5A2	CDCA5	SET	MTA2	ETS2
DIAPH3	MYBL1	ILF3	ATG3	CENPV	BANF1	LIMA1
GADD45A	TBCE	MCM7	RBM22	CDCA8	SSR3	PLAU
TIMELESS	CSE1L	MAD2L1BP	DNAJA3	POLE3	PRMT5	PTGER2
TK1	AKAP12	KIF2C	KIF23	GRPEL1	SOCS2	GJA1
MUTYH	CRY1	NUP85	NUP88	CEP57	NEIL2	MYC
IL12A	SEH1L	KATNB1	DTYMK	NUF2	NXT1	F3
CEP290	POLH	SGO1	TOP2A	KIF18B	JADE1	IGFBP3
TUBG1	MCM3	NUP50	GINS1	CRIM1	TIMM10	EDN1
HAT1	TAF5L	CEP55	XPO5	PSMC3IP	HAUS4	

Fig. S14. Hippo pathway inhibition did not upregulate the YAP oncogenic program

RNAseq data analysis indicated that, among 134 YAP/TAZ/TEAD target genes involved in cell proliferation[15], 132 were expressed in 786-O cells. Among these genes, only 16 were upregulated (green) whereas 18 were downregulated (red) in 786-O cells treated with XMU-MP-1 compared with vehicle treated cells. Most of these genes (98) were not significantly changed in their expression levels after XMU-MP-1 treatment.

Reference

13 Cho, Y. S. *et al.* Regulation of Yki/Yap subcellular localization and Hpo signaling by a nuclear kinase PRP4K. *Nature communications* **9**, 1657 (2018).

14 Reddy, T. E. *et al.* Genomic determination of the glucocorticoid response reveals unexpected mechanisms of gene regulation. *Genome Res* **19**, 2163-2171 (2009).

15 Zanconato, F. *et al.* Genome-wide association between YAP/TAZ/TEAD and AP-1 at enhancers drives oncogenic growth. *Nat Cell Biol* **17**, 1218-1227 (2015).
